# Supplementary material for: The developing Human Connectome Project (dHCP) automated resting-state functional processing framework for newborn infants
Source: Neuroimage. 2020 Dec;223:117303. doi: 10.1016/j.neuroimage.2020.117303 (PMC7762845; doi:10.1016/j.neuroimage.2020.117303)
Supplement: Supplementary file 1 [file mmc1.pdf]

9. Supplementary

9.1. Fieldmap comparison

Visual inspection identified that 12.7% (75 of 590) spin-echo EPI in the dHCP data was contaminated in all volumes, such that picking the best pair of spin-echo EPI volumes was inadequate. In this circumstance, the fall-back procedure was to use the dual-echo-time-derived fieldmap instead of the spin-echo-EPI-derived fieldmap. To ensure that the dual-echo-time and spin-echo-EPI derived fieldmaps could be used interchangeably, we evaluated the similarity between the two. For each subject the spin-echo-EPI and dual-echo-time fieldmaps were resampled to the native functional space and then converted to a voxel displacement/shift map using FSL FUGUE. The shift maps were then masked by an eroded brain mask, to avoid edge effects as a consequence of registration misalignment. Supplementary Figure 3 presents an example dual-echo-time and spin-echo-EPI derived fieldmap from a single subject, as well as the distribution of voxel displacements for all in-brain voxels from 409 subjects that had good quality dual-echo-time and spin-echo-EPI derived fieldmaps. The single-subject fieldmaps look qualitatively similar, although the dual-echo-time-derived fieldmap appears smoother and the spin-echo-EPI-derived fieldmap appears to have greater values. This is supported by the voxel displacement distribution where the spin-echo-EPI-derived fieldmap has a slightly greater mean voxel displacement than the dual-echo-time-derived fieldmap. Two factors likely contribute to this difference, 1) the dual-echo-time fieldmap was acquired at lower resolution than the spin-echo-EPI (3x3x6mm and 2.15mm isotropic respectively), and 2) the dual-echo-time-derived fieldmap was low-pass filtered as part of the reconstruction process. Furthermore, the distribution of the voxel-wise difference between the dual-echo-time and spin-echo-EPI displacement/shift maps shows that 95% of voxels differ by less than 1 voxel shift and 99% by less than two voxels. We also inspected the distribution of spatial correlation between dual-echo-time and spin-echo-EPI derived fieldmaps across subjects, and observed good correspondence with 75% (i.e., 25th percentile) of subjects showing correlation > 0.6. Given that the ground truth is unknown and that both the dual-echo-time and spin-echo-EPI derived fieldmaps are qualitatively and quantitatively similar, we felt justified in using the dual-echo-time-derived fieldmap as a back-up in cases where the spin-echo-EPI-derived was excessively contaminated by movement.

9.2. Quality control prior to the dHCP neonatal fMRI pipeline

The following is adapted from the release notes for the second dHCP data release. For further information see: http://www.developingconnectome.org/release-notes/

QC on the released dHCP data is performed at numerous stages in the analysis, including within the dHCP neonatal fMRI pipeline as described in section 3.6. There are three QC stages implemented prior to the neonatal fMRI pipeline:

1) Scanning notes were recorded by the radiographers, and failed scans were manually flagged as pass/fail depending on if the issue affects the fMRI
2) After reconstruction the images were visually inspected and each image was flagged as PASS/FAIL
3) The structural pipeline QC combined several sources of information: 479 scans were scored visually as part of an atlas construction project – we excluded scans with more than minor motion artifacts in T2. We excluded 11 scans we knew to be in error. We excluded scans on which the structural pipeline failed to run, or on which the separate structural QC pipeline failed to run. We did a visual inspection of all white matter surfaces and excluded one scan that was obviously failing.

The dHCP-538 cohort used within this paper comprises 538 subjects that passed all three stages of QC prior to the fMRI pipeline.

9.3. Detailed registration methods

Primary registrations:

1. *fieldmap-to-structural*: rigidly align the derived fieldmap magnitude image (see Section 3.2) to the native structural T2w space using FSL FLIRT (Jenkinson et al., 2002b; Jenkinson and Smith, 2001). A boundary-based registration (BBR) (Greve and Fischl, 2009) cost function is used if the fieldmap was derived from the spin-echo EPI using TOPUP. However, the correlation ratio cost function is used if the fieldmap was derived from the gradient-echo, because the magnitude image lacked sufficient anatomical detail for BBR. The fieldmap-to-structural transform is then applied to re-sample the fieldmap image into the native structural space.
2. *sbref-to-structural*: rigidly align the single-band EPI image (sbref) with the native structural T2w space and correct for susceptibility distortions in the sbref using FSL FLIRT, with the BBR cost function, and FSL FUGUE. This step requires the fieldmap to be in the native structural space (calculated in the previous registration stage) to correct for susceptibility distortions in the sbref.
3. *functional-to-sbref (distorted)*: rigidly align the functional multiband EPI image with the sbref using FSL FLIRT with the default correlation ratio cost function. This registration step is performed prior to susceptibility distortion correction of the functional multiband EPI as described in Section 3.4, therefore both the functional multiband EPI image and the sbref will contain susceptibility distortions. The first volume of the functional multiband EPI is used as the source (moving) image in this registration because the subsequent motion correction and distortion correction stage defines the functional space from the first volume (see Section 3.4).
4. *functional-to-sbref (undistorted)*: after motion correction and distortion correction, rigidly align the distortion-corrected functional multiband EPI image with the distortion-corrected sbref using FSL FLIRT with the default correlation ratio cost function. All volumes in the corrected functional multiband EPI image are aligned as consequence of the motion correction, therefore the temporal mean is used as the source (moving) image in this registration as it typically has superior SNR compared to a single volume.
5. *template-to-structural*: align the structural image to the dHCP volumetric template (Schuh et al., 2018). Template-to-structural registration is performed with a multi-modal non-linear registration (ANTs SyN)(Avants et al., 2008) of the age-matched T1w and T2w template to the subject's T1w and T2w structural, which is then combined with the appropriate atlas week-to-week transforms to yield a (40 week) template-to-structural transform. We also evaluated FSL FNIRT (Jenkinson et al., 2012) and MIRTK Register (Similarity+Affine+FFD transformation model) (Schuh et al., 2018a) and found that Register achieved excellent alignment but was not sufficiently regularised, resulting in inversion inaccuracy, whilst FNIRT was well regularised but did not produce alignments with sufficient accuracy (data not shown). We expect that good results could be achieved with both tools if their parameters were optimised, however ANTs SyN provided a good trade-off between alignment and regularisation with minimal parameterisation. In the event that the age of the subject is outside the range covered by the atlas, the closest template age within the atlas is used. Furthermore, some subjects do not have a T1w image, so in this instance only the T2w is used.

Composite registrations:

1. *fieldmap-to-functional*: constructed by combining the fieldmap-to-structural transform with the inverse sbref-to-structural and inverse functional-to-sbref (distorted) transforms. This allows for the fieldmap to be resampled to the native functional space, which is essential for the subsequent motion correction and distortion correction (Section 3.4). We have found that aligning the fieldmap with the functional via the structural is very robust and precise, largely because both sub-steps use BBR cost functions.
2. *functional-to-structural (undistorted):* constructed by combining the functional-to-sbref (undistorted) affine with the sbref-to-structural affine, which yields a linear transform that aligns the motion and distortion corrected functional multiband EPI with structural T2w.
3. *functional-to-template (undistorted):* constructed by combining the functional-to-structural (undistorted) transform with the inverse template-to-structural transform to yield the functional-to-template (undistorted) non-linear transform to align the motion and distortion corrected functional multiband EPI with the 40-week dHCP template space with a single resampling.

9.4. Frame censoring

A popular and effective method of dealing with head motion is using spike regression (Satterthwaite et al., 2012) or scrubbing (Power et al., 2014, 2012), collectively referred to as frame censoring. We evaluated frame censoring as an alternative to ICA+FIX denoising.

Both spike regression and scrubbing first identify time-points (whole volumes referred to as frames) and then censor these frames so that they do not affect downstream analysis. The methods differ in how they identify the contaminated frames and how they censor the contaminated frames (Parkes et al., 2018). Both methods use framewise displacement (FD; see Table 3) with a fixed displacement threshold to identify contaminated frames. Scrubbing additionally uses DVARS (see Table 3), also with a fixed threshold. Censoring in spike regression is achieved by creating a nuisance regressor per contaminated frame, whereas scrubbing either excludes contaminated frames and/or replaces contaminated frames with surrogate data depending upon what is appropriate for downstream processing. Additionally, both techniques employ a heuristic that discards entire subjects if there are insufficient uncontaminated time-points.

Frame censoring methods can be expensive in terms of the number of volumes censored, particularly in high-motion cohorts such as neonates. This is particularly true for the dHCP because the babies are scanned without sedation. Using framewise displacement (Power et al., 2012) as a surrogate for head motion and a threshold of 0.25 mm, as advocated by Satterthwaite et al. (2013), results in ~20% of frames being flagged as motion corrupted. Furthermore, if we exclude subjects with < 4 minutes of uncorrupted data, the minimum recommended in spike regression and scrubbing (Parkes et al., 2018; Power et al., 2014; Satterthwaite et al., 2013), then 148 subjects are excluded. Thus, to implement frame censoring we needed to relax these criteria. To identify contaminated frames we used DVARS (post motion and distortion correction), with a threshold defined as the 75th percentile + 1.5 times the inter-quartile range (the outlier whisker when creating boxplots). This resulted in 6.5% of frames being flagged as outliers. We did not implement a minimum duration of uncorrupted data. To achieve censoring, we simply removed the contaminated volumes, because the downstream analysis plan was to perform group sICA which is not sensitive the discontinuous time.

Frame-censoring was evaluated on the 512 subjects from that dHCP-538 data that passed QC (see Section 3.6). Frame censored data was compared to the ICA+FIX denoised data (described in Section 3.5) using spatial and network matrix similarity to the unbiased group template (also described in Section 3.5). This required running a low-dimension group ICA (dimension=25) across all data (all subjects, frame-censored and ICA+FIX denoised) to generate unbiased group spatial maps. The unbiased group maps were visually inspected and 12 RSN consistent maps identified (see Supplementary Figure 4).

Group paired-differences in spatial and network similarity between frame-censored and ICA+FIX denoised groups were calculated using FSL RANDOMISE (Winkler et al., 2014) with 5000 permutations. Multiple comparison correction was achieved by FDR correction. ICA+FIX denoised data resulted in significantly ($p<0.025$) greater spatial similarity to 10 of the 12 unbiased group RSN maps, whilst frame censoring only showed significantly ($p<0.025$) greater spatial similarity for one of the unbiased group RSN maps (see Supplementary Figure 5). Furthermore, ICA+FIX denoising resulted in significantly ($p<0.025$) greater network matrix similarity to the unbiased group network matrix than frame censoring (see Supplementary Figure 6).

These results suggest, that in this context, with this specific variant of frame censoring, that ICA+FIX denoising performs better than frame censoring on these specific benchmarks. However, ICA+FIX has the added advantage that it is able correct for confounds beyond motion, such as multi-band artefacts, scanner artefacts, venous/arterial related artefacts, and spin-history effects. Thus, given this evidence, we have opted to implement ICA+FIX in this dHCP neonatal fMRI pipeline which enables us to largely mitigate the effects of many confounds without excluding any subjects or time-points. Furthermore, we have avoided introducing a hard-censoring step at an intermediate processing point, which could have ramifications for downstream processing. It is important to note that this analysis was intended to provide readers with a sense for how the results from the two techniques compare, but does not necessarily provide definitive results regarding the performance of the two approaches in all situations, with all possible settings and analysis decisions included, as we only consider a single robust implementation of frame censoring and a specific set of outcome metrics. We expect both approaches could be valuable and effective for infant fMRI processing in the right context, and both likely better than doing neither.

9.5. RSN development with age

Before regressing the RSNs on age to look for developmental changes, we examined a number of age-related confounds. Specifically, we correlated DVARS and FD (as surrogates for motion), tSNR, and brain volume (estimated as the number of voxels in the subject's brain mask in func space). We observed a strong positive correlation of brain volume with age (r=0.86), a small positive correlation of mean DVARS (r=0.05) and mean FD (r=0.16) with age, and a small negative correlation of tSNR (r=-0.15) with age (see Supplementary Figure 7). Movement and tSNR are clear confounds that we wish to control for, however brain volume is more challenging because it can be both a confound (due to differences in relative resolution and signal) and a legitimate feature of development. Here we control for brain volume and present RSN correlations with development.

We used FSL dual-regression (DR)(Nickerson et al., 2017) to regress all the PFM-group-maps onto the individual subject fMRI to yield subject-specific time-courses and spatial maps. As recommended by Nickerson et al. (2017), when performing DR the subject-specific time-courses were variance normalised before the second stage of DR which means that the single-subject DR spatial maps capture both the spatial distribution of the network (i.e. "shape") as well as the "amplitude" of the network activity. To allow us to delineate the contribution of just amplitude alone, we additionally calculated the median absolute deviation of the DR time-courses (not variance-normalised) as an estimate of amplitude. To investigate changes with age, we regressed the spatial maps and amplitudes on age, controlling for DVARS, FD, tSNR, and brain volume (see Supplementary Figure 8) using FSL RANDOMISE (Winkler et al., 2014) with 5000 permutations. Multiple comparison correction was achieved by FDR correction with a threshold of 0.05.

The DR spatial maps show a significant effect for age in all modes, in voxels that are spatially consistent with the group PFM map. Furthermore, the DR amplitudes show a significant increase in network amplitudes with age for all modes, which indicates that the age effects are, at least partially, driven by this increased amplitude of network activity.

9.6. Supplementary references

Arichi, T., Fagiolo, G., Varela, M., Melendez-Calderon, A., Allievi, A., Merchant, N., Tusor, N., Counsell, S.J., Burdet, E., Beckmann, C.F., Edwards, A.D., 2012. Development of BOLD signal hemodynamic responses in the human brain. NeuroImage 63, 663–673. https://doi.org/10.1016/j.neuroimage.2012.06.054

Avants, B.B., Epstein, C.L., Grossman, M., Gee, J.C., 2008. Symmetric diffeomorphic image registration with cross-correlation: Evaluating automated labeling of elderly and neurodegenerative brain. Med. Image Anal. 12, 26–41. https://doi.org/10.1016/j.media.2007.06.004

Greve, D.N., Fischl, B., 2009. Accurate and robust brain image alignment using boundary-based registration. NeuroImage 48, 63–72. https://doi.org/10.1016/j.neuroimage.2009.06.060

Jenkinson, M., Bannister, P., Brady, M., Smith, S., 2002b. Improved Optimization for the Robust and Accurate Linear Registration and Motion Correction of Brain Images. NeuroImage 17, 825–841. https://doi.org/10.1006/nimg.2002.1132

Jenkinson, M., Smith, S., 2001. A global optimisation method for robust affine registration of brain images. Med. Image Anal. 5, 143–156

Nickerson, L.D., Smith, S.M., Öngür, D., Beckmann, C.F., 2017. Using Dual Regression to Investigate Network Shape and Amplitude in Functional Connectivity Analyses. Front. Neurosci. 11. https://doi.org/10.3389/fnins.2017.00115

Parkes, L., Fulcher, B., Yücel, M., Fornito, A., 2018. An evaluation of the efficacy, reliability, and sensitivity of motion correction strategies for resting-state functional MRI. NeuroImage 171, 415–436.

Power, J.D., Barnes, K.A., Snyder, A.Z., Schlaggar, B.L., Petersen, S.E., 2012. Spurious but systematic correlations in functional connectivity MRI networks arise from subject motion. NeuroImage 59, 2142–2154 https://doi.org/10.1016/j.neuroimage.2011.10.018

Power, J.D., Mitra, A., Laumann, T.O., Snyder, A.Z., Schlaggar, B.L., Petersen, S.E., 2014. Methods to detect, characterize, and remove motion artifact in resting state fMRI. NeuroImage 84. https://doi.org/10.1016/j.neuroimage.2013.08.048

Satterthwaite, T.D., Wolf, D.H., Loughead, J., Ruparel, K., Elliott, M.A., Hakonarson, H., Gur, R.C., Gur, R.E., 2012. Impact of in-scanner head motion on multiple measures of functional connectivity: Relevance for studies of neurodevelopment in youth.

Schuh, A., Makropoulos, A., Robinson, E.C., Cordero-Grande, L., Hughes, E., Hutter, J., Price, A.N., Murgasova, M., Teixeira, R.P.A.G., Tusor, N., Steinweg, J.K., Victor, S., Rutherford, M.A., Hajnal, J.V., Edwards, A.D., Rueckert, D., 2018a. Unbiased construction of a temporally consistent morphological atlas of neonatal brain development. bioRxiv 251512. https://doi.org/10.1101/251512

Winkler, A.M., Ridgway, G.R., Webster, M.A., Smith, S.M., Nichols, T.E., 2014. Permutation inference for the general linear model. NeuroImage 92, 381–397. https://doi.org/10.1016/j.neuroimage.2014.01.060

9.7. Supplementary Figures

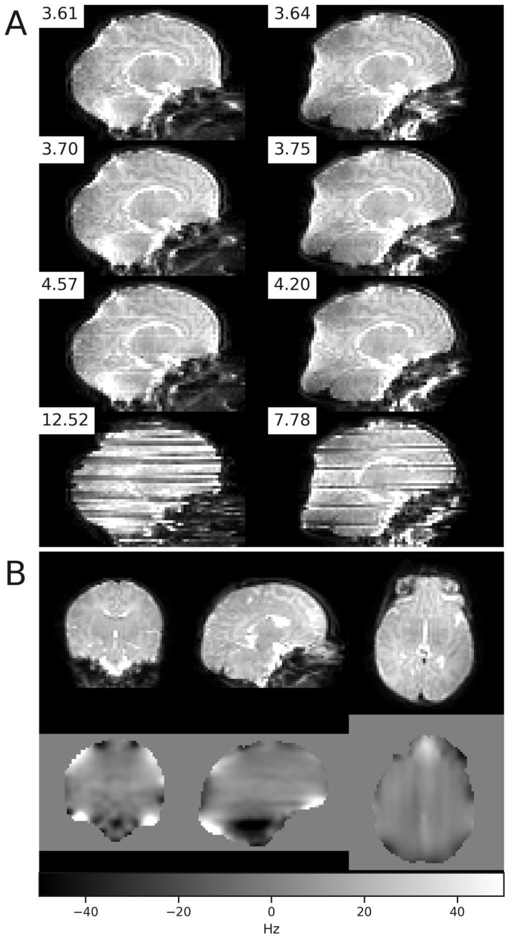

Supplementary Figure 1. (A) Eight volumes of the spin-echo EPI from a single subject with AP (left) and PA (right) phase encode directions. Z-smoothness scores are presented with each volume. The two volumes in the last row have stereotyped striping artefact due to subject movement, resulting in higher z-smoothness scores. The two volumes in the first row were selected as the two "best" images based on z-smoothness. (B) Motion and distortion corrected spin-echo EPI (upper) and estimated susceptibility-induced off-resonance field (lower) derived from the spin-echo EPI in (A) using FSL TOPUP.

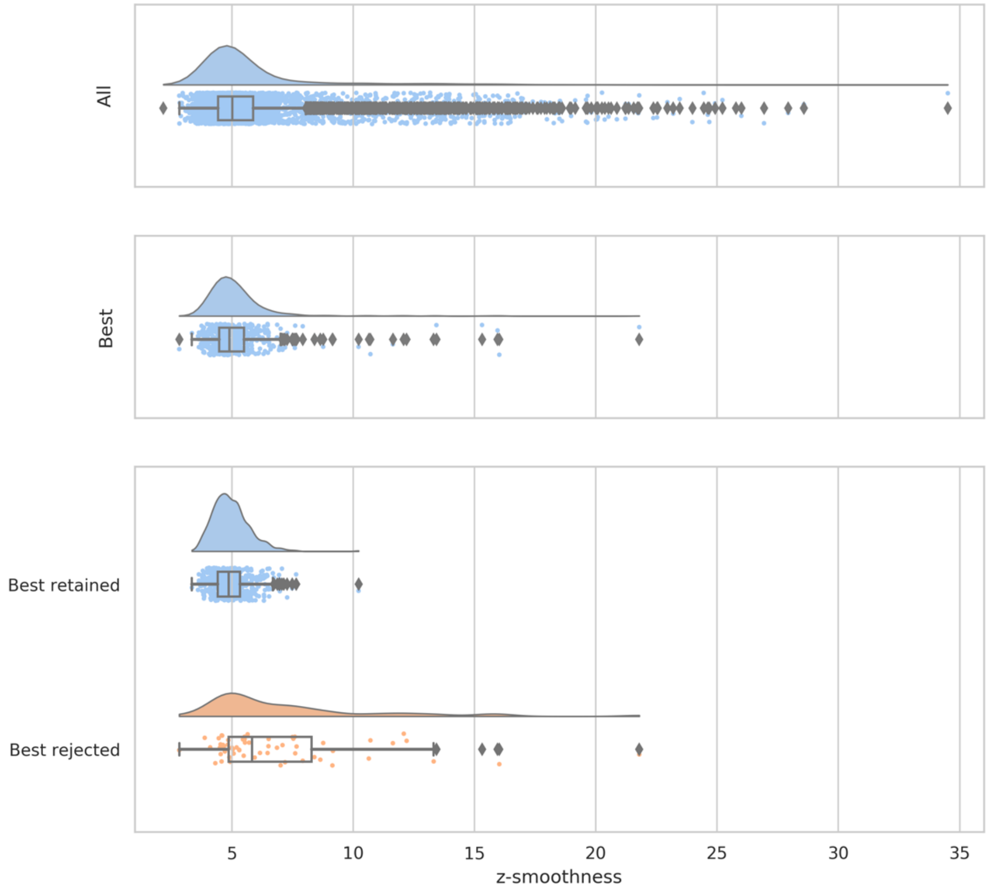

Supplementary Figure 2. (Upper) Distribution of z-smoothness across all spin-echo-EPI volumes (8 per subject) across all subjects (N=538). (Middle). Distribution of z-smoothness for selected "best" quality spin-echo EPI volume across subjects. Best is defined by, first, picking the best pair of spin-echo-EPI volumes (one per phase encode direction) that had the lowest z-smoothness within subject, and then using the maximum z-smoothness value from that pair. (Lower) Same distribution as the middle plot but split by whether all spin-echo-EPI volumes were retained or rejected by visual inspection.

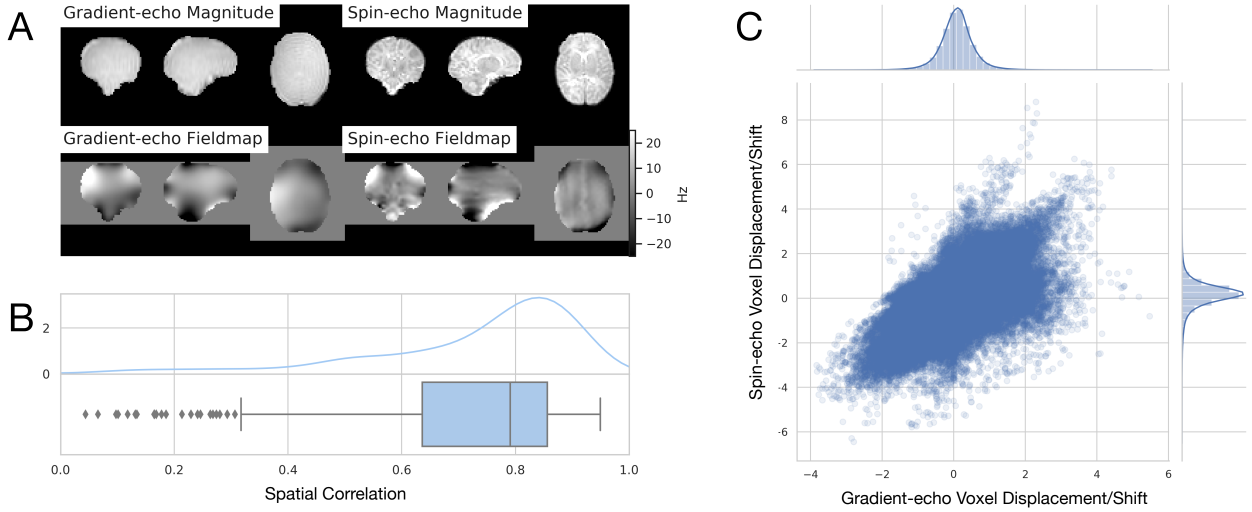

Supplementary Figure 3. (A) Exemplar gradient-echo and spin-echo derived fieldmaps and magnitude from a single-subject. The spatial correlation of the two fieldmaps is 0.71. (B) Distribution of spatial correlation between gradient-echo and spin-echo fieldmaps from 409 subject. (C) Distribution of voxel displacement/shift for in-brain voxels from 409 subjects. To improve visualisation of the scatter plot density, only a 10% random sample of the (>11 million) voxel displacements/shifts are plotted.

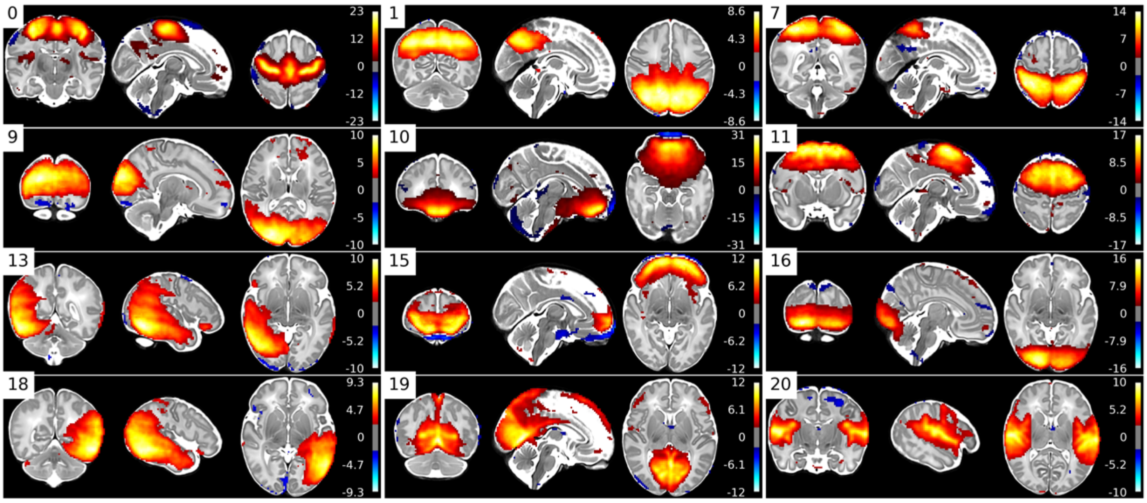

Supplementary Figure 4. Unbiased group RSN template maps created from ICA+FIX and frame-censored data.

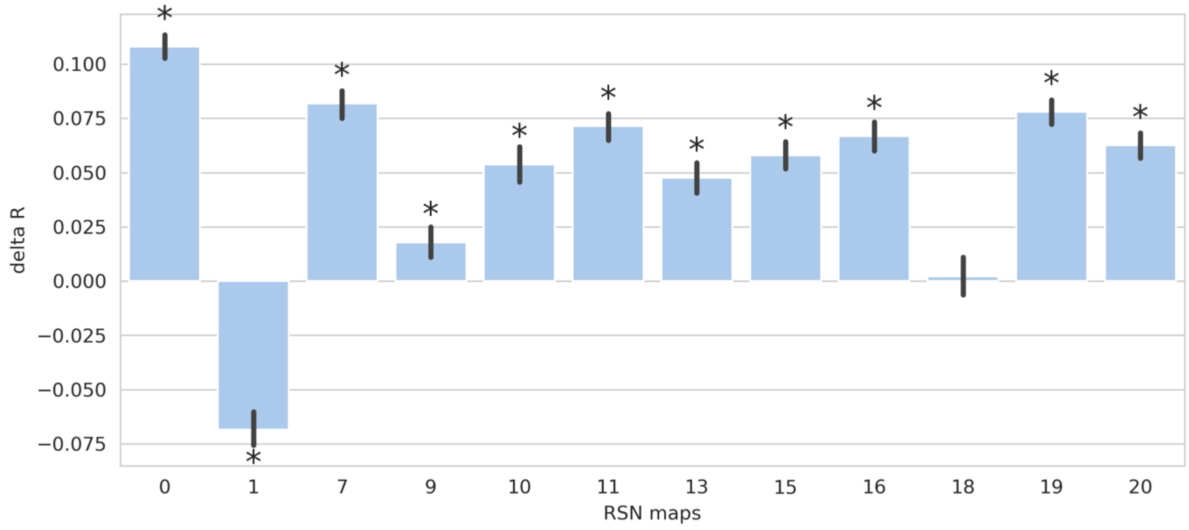

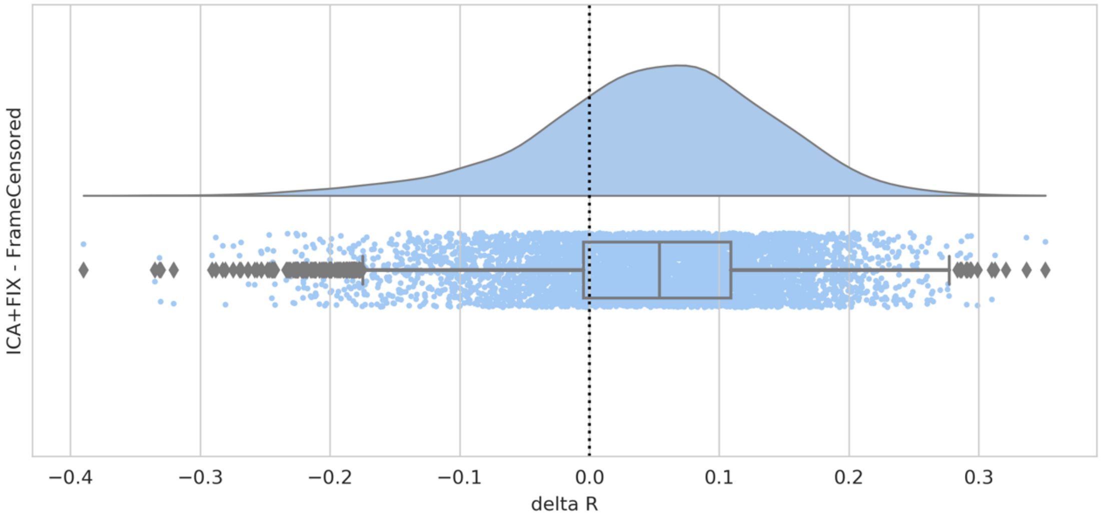

Supplementary Figure 5. (Upper) Mean paired-difference (ICA+FIX minus Frame Censored) of spatial similarity to the unbiased group template per map. Asterisks indicate significant differences. (Lower) Distribution of paired differences (ICA+FIX minus Frame Censored) of spatial similarity to the unbiased group template pooled over all spatial maps.

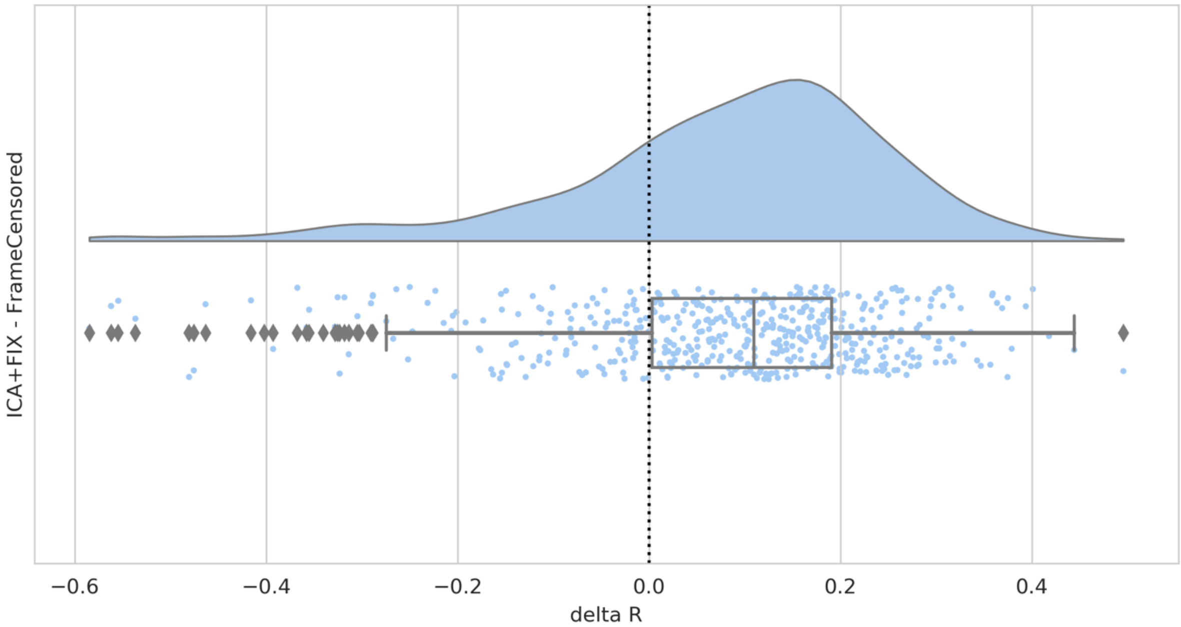

Supplementary Figure 6. Distribution of paired differences (ICA+FIX minus Frame Censored) of network matrix similarity to the unbiased group network matrix.

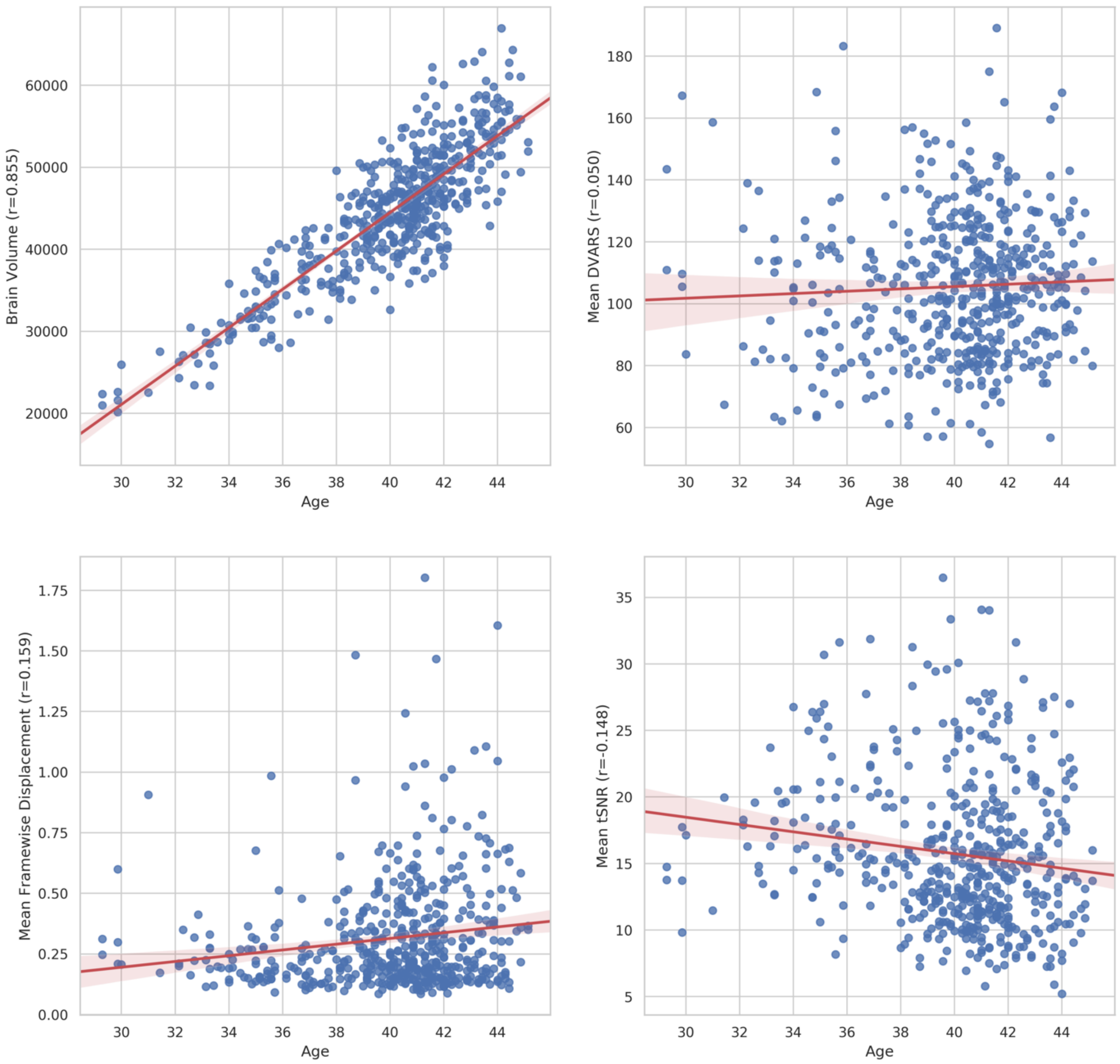

Supplementary Figure 7. Age-related confounds. Age is the post-menstrual age-at-scan in weeks.

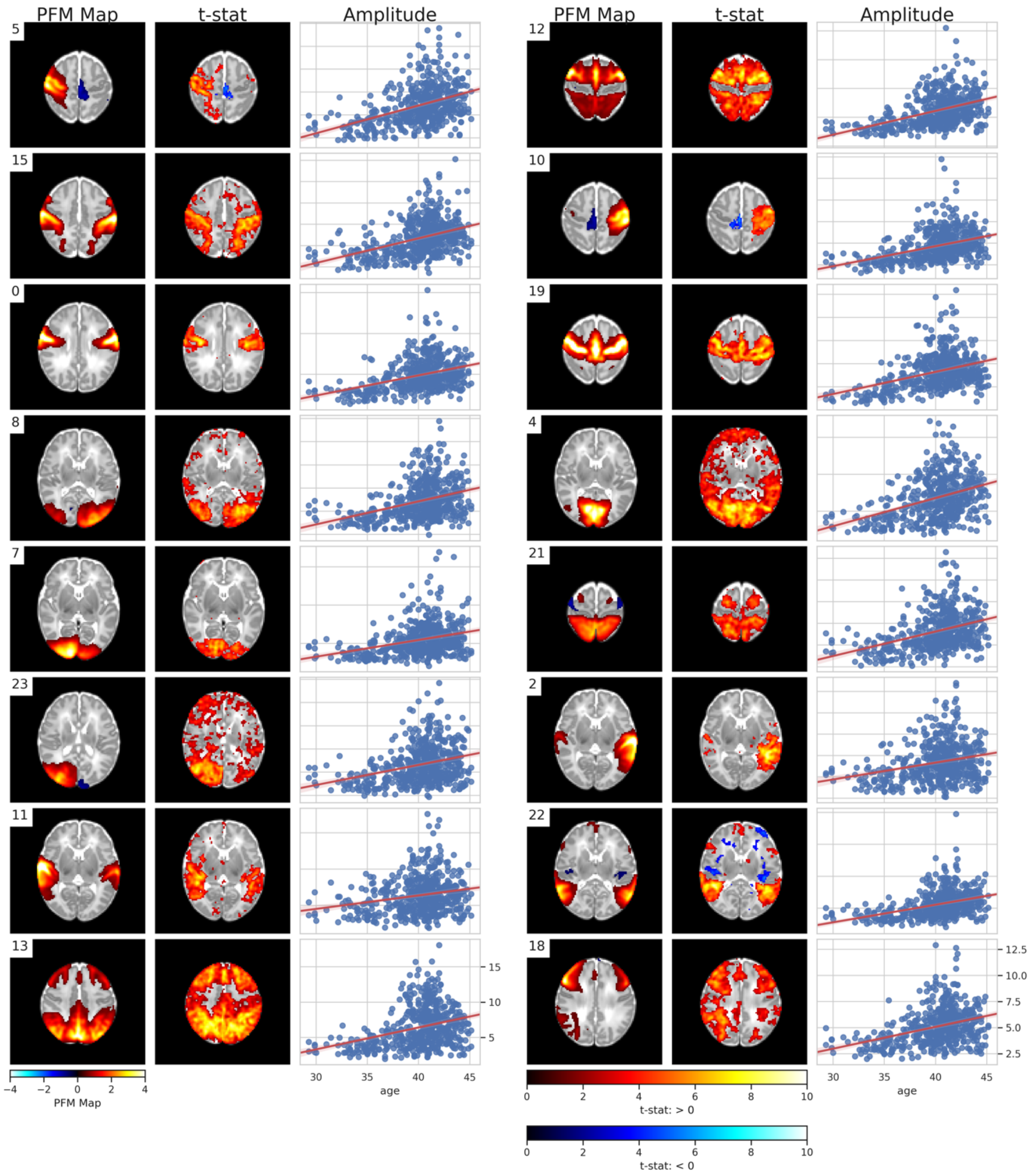

Supplementary Figure 8. PROFUMO group spatial maps (PFM Map), t-statistic of age regressed on the DR spatial maps (t-stat), and DR amplitudes with age (Amplitude) for the 16 modes qualitative assessed as corresponding to adult resting-state networks. Age is the post-menstrual age-at-scan in weeks. Brain volume, mean DVARS, mean tSNR, and mean FD confounds are controlled. Only significant results are shown. Multiple comparison correction was achieved by FDR correction with a 0.05.

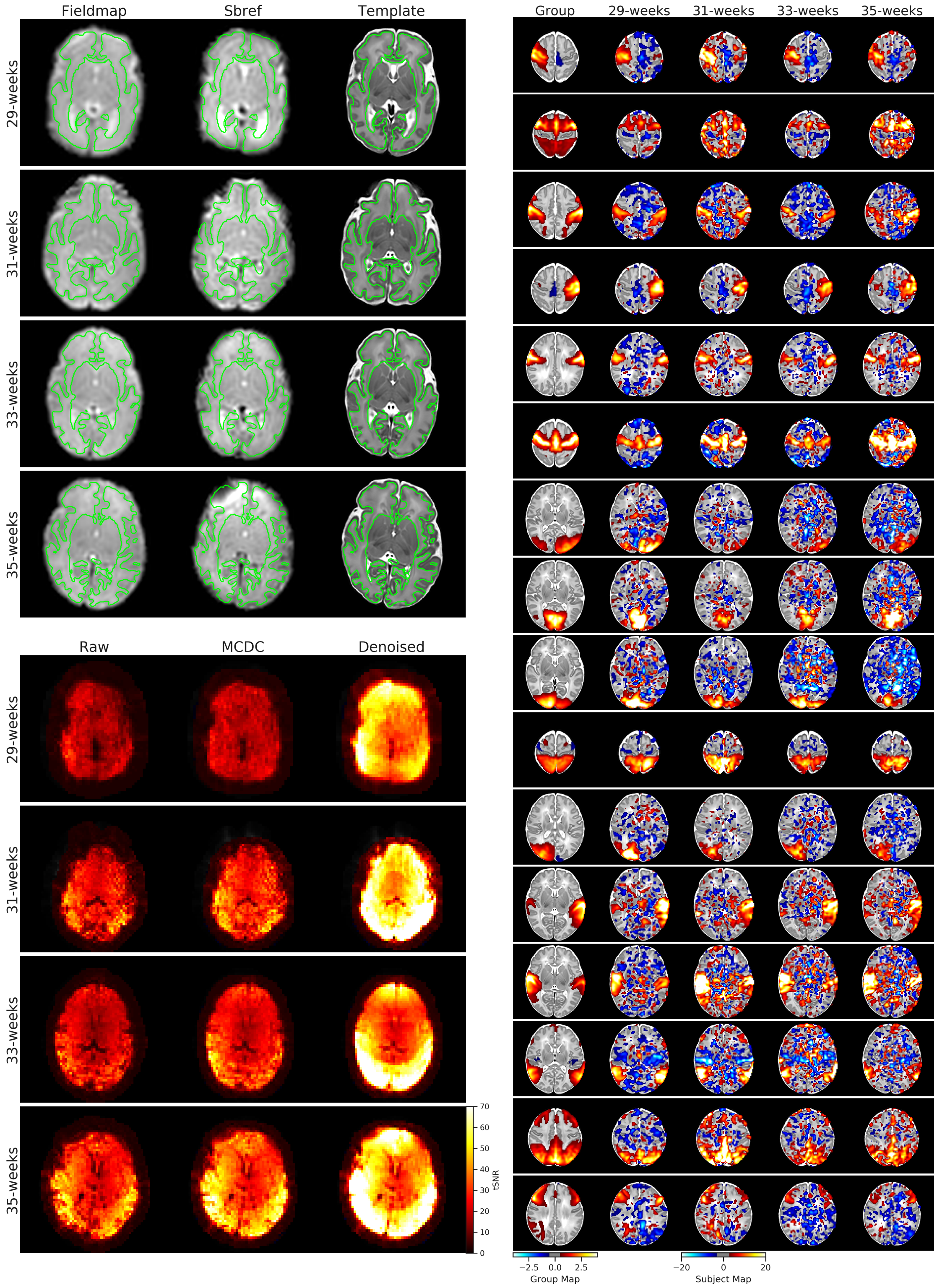

Supplementary Figure 9. Examples of pre-term subjects scanned at 29-weeks (born at 28-weeks), 31-weeks (born at 30-weeks), 33-weeks (born at 31-weeks), and 35-weeks (born at 34-weeks). Upper left images demonstrate registration quality for these subjects. Fieldmap, sbref, and template images are resampled to the native structural reference space. The outline of the native structural white matter discrete segmentation is overlaid in green. Lower left images demonstrate mean tSNR for these pre-term subjects at different pipeline stages: raw EPI, motion and distortion corrected EPI (MCDC), and denoised EPI. The right images demonstrate the single-subject spatial maps for these subjects after dual-regressing 16 profumo group RSN maps onto them. The first column is the group map, and the subsequent columns are the dual-regressed single subject maps.

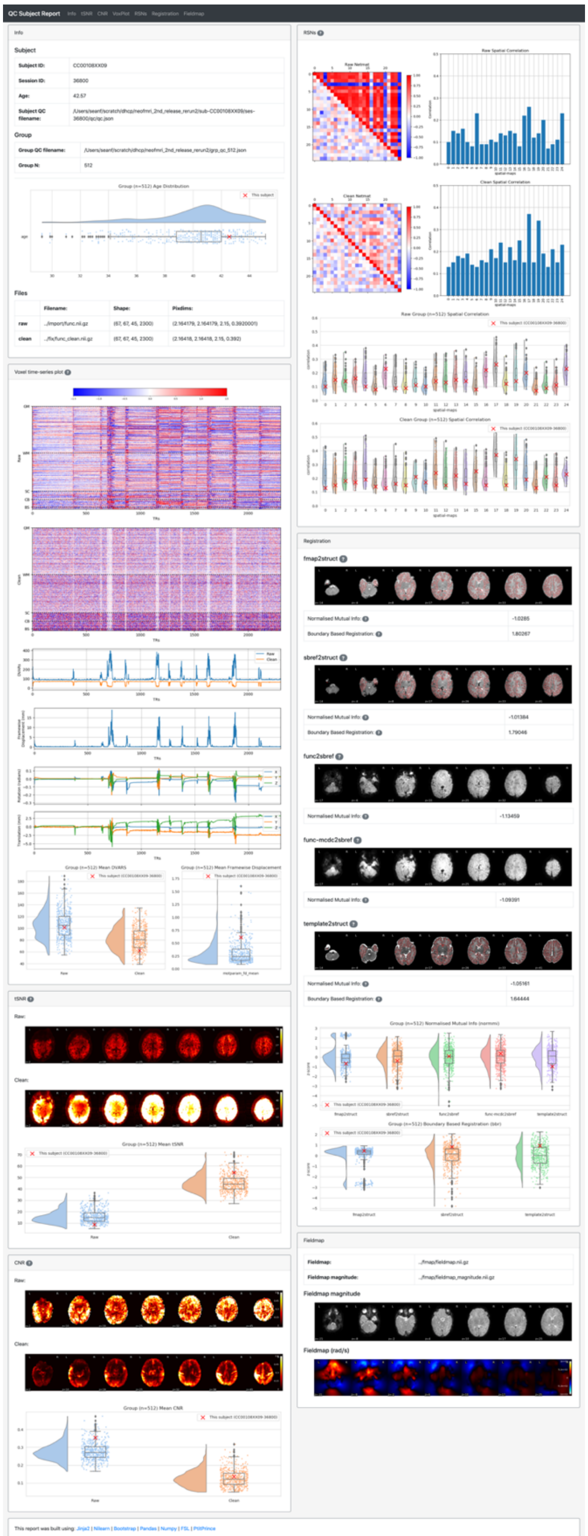

Supplementary Figure 10. Screen shot of automated QC report for a single subject. The dHCP neonatal fMRI pipeline automatically calculates a number of QC metrics (MP, DVARS, FD, tSNR, CNR, NMI; see Table 3) and generates this HTML QC report for each subject. The report presents each QC metric for the individual within the context of the group distribution for the corresponding metric. Additionally, the report also presents descriptive/qualitative summaries of the subject's data quality in the form of "voxplots" and spatial maps for each metric as applicable. The report generation tool utilises a variety of open source packages including Jinja2 (http://jinja.pocoo.org/docs/2.10/), Bootstrap (https://getbootstrap.com/), Pandas (https://pandas.pydata.org/), Numpy (https://www.numpy.org/), Seaborn (https://seaborn.pydata.org), Nilearn (http://nilearn.github.io/), FSL (https://fsl.fmrib.ox.ac.uk/fsl/fslwiki), and PtitPrince (https://github.com/pog87/PtitPrince).

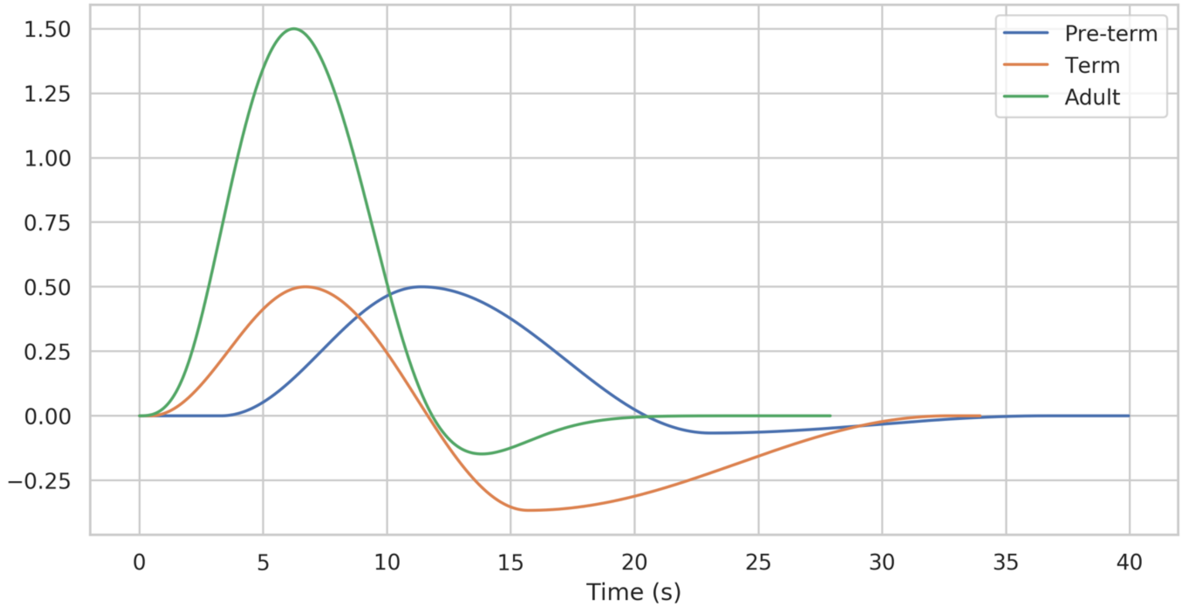

Supplementary Figure 11. Term and pre-term HRF models constructed for this study, and the default adult HRF model within PROFUMO. The term and pre-term haemodynamic response characteristics are adapted from Arichi et al. (2012). The amplitude of each HRF is rescaled as part of the fitting process, however, for visualisation purposes the peak amplitudes here have been scaled to be consistent with the measured amplitudes in Arichi et al. (2012).
